# Artificial intelligence breast ultrasound and handheld ultrasound in the BI-RADS categorization of breast lesions: A pilot head to head comparison study in screening program

**DOI:** 10.3389/fpubh.2022.1098639

**Published:** 2023-01-18

**Authors:** Xiaoxi Huang, Youhui Qiu, Fangfang Bao, Juanhua Wang, Caifeng Lin, Yan Lin, Jianhang Wu, Haomin Yang

**Affiliations:** ^1^Department of Breast, Fujian Maternity and Child Health Hospital, College of Clinical Medicine for Obstetrics and Gynecology and Pediatrics, Fujian Medical University, Fuzhou, Fujian, China; ^2^Department of Surgery, Changting Maternity and Children's Hospital, Longyan, Fujian, China; ^3^Department of Gynecology, Changting Maternity and Children's Hospital, Longyan, Fujian, China; ^4^Department of Anesthesiology, Changting Maternity and Children's Hospital, Longyan, Fujian, China; ^5^Department of Public Health, Changting Maternity and Children's Hospital, Longyan, Fujian, China; ^6^Department of Ultrasound, Fujian Maternity and Child Health Hospital, College of Clinical Medicine for Obstetrics and Gynecology and Pediatrics, Fujian Medical University, Fuzhou, Fujian, China; ^7^Department of Epidemiology and Health Statistics, School of Public Health, Fujian Medical University, Fuzhou, Fujian, China

**Keywords:** artificial intelligence, breast ultrasound, agreement, breast cancer, cancer screening (MeSH)

## Abstract

**Background:**

Artificial intelligence breast ultrasound diagnostic system (AIBUS) has been introduced as an alternative approach for handheld ultrasound (HHUS), while their results in BI-RADS categorization has not been compared.

**Methods:**

This pilot study was based on a screening program conducted from May 2020 to October 2020 in southeast China. All the participants who received both HHUS and AIBUS were included in the study (*N* = 344). The ultrasound videos after AIBUS scanning were independently watched by a senior radiologist and a junior radiologist. Agreement rate and weighted Kappa value were used to compare their results in BI-RADS categorization with HHUS.

**Results:**

The detection rate of breast nodules by HHUS was 14.83%, while the detection rates were 34.01% for AIBUS videos watched by a senior radiologist and 35.76% when watched by a junior radiologist. After AIBUS scanning, the weighted Kappa value for BI-RADS categorization between videos watched by senior radiologists and HHUS was 0.497 (*p* < 0.001) with an agreement rate of 78.8%, indicating its potential use in breast cancer screening. However, the Kappa value of AIBUS videos watched by junior radiologist was 0.39, when comparing to HHUS.

**Conclusion:**

AIBUS breast scan can obtain relatively clear images and detect more breast nodules. The results of AIBUS scanning watched by senior radiologists are moderately consistent with HHUS and might be used in screening practice, especially in primary health care with limited numbers of radiologists.

## Introduction

Breast cancer is the most common malignant tumor in women worldwide, and the incidence is increasing every year (1). As only a small fraction of breast cancer can be attributed to the known life-style risk factor (2), early detection is key to improving survival and reducing mortality associated with breast cancer (3). Mammography is widely used for breast cancer screening, whereas ultrasound is used as a supplementary screening method for women with dense breasts in European and American countries (4). Ultrasound is also recommended for breast cancer screening in primary health care in China, especially in the rural areas (5), because of the absence of ionizing radiation, its cost-effectiveness, and a lower rate of missed diagnosis with dense breasts (6).

However, handheld ultrasound (HHUS) requires working experience of the radiologists, and it is difficult to standardize (7). Therefore, several artificial intelligence methods have been applied in breast ultrasound (8). The Artificial Intelligence Breast Ultrasound Diagnostic System (AIBUS) was developed in 2018 and used in the breast cancer screening programs of several provinces in China since 2019, with a similar detection rate of breast nodules and shorter examination time as compared to HHUS (9, 10). AIBUS can automate and standardize breast scanning and video acquisition procedures without a radiologist. The ultrasound videos are transmitted to the server immediately, and an artificial intelligence algorism is used to provide suspected lesion videos for multiple users and multiple terminals to make the diagnosis. It can also longitudinally store the ultrasound video and provide long-term breast health surveillance for women. The mobility of AIBUS may further increase the accessibility of the breast cancer screening program and address the shortage of radiologists and equipment in the primary care settings in rural and remote areas.

Despite the various comparative studies on other operator-independent automated breast scanners and HHUS (11), the agreement of Breast Imaging Reporting and Data System (BI-RADS) score categorization between AIBUS and HHUS has not been evaluated. In addition, as radiologists finally watched the videos from AIBUS and decided the BI-RADS score, it is unclear whether radiologists' experience might influence the agreement between AIBUS and HHUS.

This study aimed to analyse the agreement between AIBUS and HHUS among women who underwent both examinations and to determine the effect of radiologists' experience on categorizing BI-RADS scores.

## Methods

### Study population

We recruited women who participated in the breast cancer screening program in Changting in southeast China between May 2020 and October 2020. As required by the breast cancer screening program in rural China, ultrasound was the primary screening approach and these women had not received any breast examination for at least 3 years. We included those who successively underwent both HHUS and AIBUS examination, resulting in a total of 344 women aged 35–63 years old. The study was approved by the ethics committee in Fujian Maternity and Child Health Hospital and all methods were performed in accordance with relevant guidelines and regulations.

### Ultrasound examination

In HHUS examination, a commercial color Doppler ultrasound equipment (Shenzhen Mindray Bio-Medical Electronics Co., Ltd, https://www.mindray.com/en/) was used with a linear transducer (≥7.5 MHz). A comprehensive scan of the breasts was performed by senior radiologists with more than 10 years of experience to observe the position, size, shape, boundary, and internal echo of the lesions.

AIBUS was performed after HHUS using the AIBUS100 Pro artificial intelligence breast ultrasound diagnostic system (Shenzhen Hanwei Intelligent Medical Technology Co., Ltd. http://www.aisono.com/). It integrates artificial intelligence, computer visualization, and robotics, with the principle based on traditional ultrasound. The women were asked to lie on their backs, and robotic arms scanned both breasts. In the scanning process, structured light, and image recognition algorithms were first used to identify scanning areas. A trajectory planning algorithm was further used to optimize the scanning route. The scanning procedure does not require a radiologist to operate on-site, realizing fully automated standardized breast scanning. After scanning, the ultrasound videos were stored and remotely watched by a senior radiologist and a junior radiologist from other hospitals. The AIBUS system also provided indications for videos with suspected nodules.

Results of HHUS and AIBUS were classified according to ultrasound BI-RADS, developed by the American College of Radiology (12). Generally, women without breast nodules are categorized as BI-RADS level 1, while the nodules are further categorized with level 2–5 according to the characteristics of the nodules. Women with BI-RADS 2 and 3 were suggested to have a regular check-up, whereas those with BI-RADS 4 and 5 were suggested for a further biopsy.

### Statistical analysis

We used McNemar's chi-square test to compare the detection rate of breast nodules using HHUS and AIBUS. Kappa test with linear weighting was further used to compare the agreement of the BI-RADS scores from AIBUS and HHUS. SPSS25.0 statistical software was used for statistical analysis, and *P* < 0.05 indicates a statistically significant finding.

## Results

The mean age of 344 participants was 47.9 years old (SD = 6.4). Majority of the women were premenopausal (65.99%) and only one woman had received breast ultrasound previously (Table 1). Among them, 51 women were detected with breast nodules using HHUS, corresponding to a detection rate of 14.83%. After watching the AIBUS scanning videos, the senior radiologists diagnosed 117 breast nodules, while the junior radiologists diagnosed 123 nodules, corresponding to the detection rates of 34.01 and 35.76%. The detection rate of breast nodules using AIBUS were significantly higher than HHUS, which detected 20% more breast nodules (Table 2).

**Table 1:** Basic characteristics of the study population.

**Characteristics**	**No. (%)**
Age (mean and SD)	47.94 (6.41)
**Education**	
Primary school/illiterate	320 (93.02)
Secondary school	24 (6.98)
**Menopausal status**	
Pre-menopause	227 (65.99)
Post-menopause	116 (33.72)
Unsure	1(0.29)
**Age at menarche**	
< 12	82 (23.84)
12–15	69 (20.06)
>15	193 (56.10)
**Previous breast examination**	
No	343 (99.71)
Yes	1 (0.29)
**Age at firth birth**	
< 22	137 (39.83)
23–24	148 (43.03)
>25	59 (17.15)
**Breast feeding**	
No	1 (0.29)
Yes	343 (99.71)

**Table 2:** Detection rates of breast nodules using HHUS and AIBUS.

**Groups**	**Breast nodules**		**Detection rate (%)**	***χ^2^***	***P*-value**
	**No**	**Yes**			
HHUS	293	51	14.83	–	–
AIBUS by senior radiologists	227	117	34.01	64.02	< 0.001
AIBUS by junior radiologists	221	123	35.76	68.12	< 0.001

The *P*-value was calculated with McNemar's test with the continuity correction.

When categorized by BI-RADS scores, 293 women were classified into BI-RADS level 1, 32 into BI-RADS level 2, and 19 into BI-RADS level 3 using HHUS. After AIBUS scanning, 227 women were classified into BI-RADS level 1, 67 into BI-RADS level 2, and 50 into BI-RADS level 3, according to the videos watched by the senior radiologists. The observed agreement rate between HHUS and AIBUS was 78.8%, and the weighted Kappa value was 0.497 (*P* < 0.001; Table 3).

**Table 3:** Comparison between AIBUS read by senior radiologists and HHUS in BI-RADS categorization.

**BI-RADS by AIBUS**	**BI-RADS by HHUS**			**Total**	**Weighted** **Kappa**	***P*-value**
	**Level 1**	**Level 2**	**Level 3**			
Level 1	227	0	0	227	0.497	< 0.001
Level 2	40	26	1	67		
Level 3	26	6	18	50		
Total	293	32	19	344		

For the same videos watched by junior radiologists, 221 women were classified into BI-RADS level 1, 52 into BI-RADS level 2, and 71 into BI-RADS level 3. The corresponding agreement rate and weighted Kappa value were 74.4% and 0.390, respectively (*P* < 0.001; Table 4), which were lower than the Kappa value estimated from the senior radiologists.

**Table 4:** Comparison between AIBUS read by junior radiologists and HHUS in BI-RADS categorization.

**BI-RADS by AIBUS**	**BI-RADS by HHUS**			**Total**	**Weighted** **Kappa**	***P*-value**
	**Level 1**	**Level 2**	**Level 3**			
Level 1	220	1	0	221	0.390	< 0.001
Level 2	27	21	4	52		
Level 3	46	10	15	71		
Total	293	32	19	344		

## Discussion

### Key results

In this head to head comparison study, we found a significantly higher detection rate of breast nodules using AIBUS as compared to HHUS. We also observed a moderate agreement in BI-RADS categorization between HHUS and AIBUS videos watched by senior radiologists, whereas the agreement was fair when junior radiologists watched the videos.

Our analysis also showed that 22.5% (66/293) of the women without nodules detected by HHUS were categorized as BI-RADS 2 or 3 by AIBUS, suggesting that AIBUS might detect some breast nodules missed by HHUS. The missed breast nodules by HHUS had similar width and echo pattern with the concordant nodules, while some of the missed nodules did not have clear margins. As the radiologists were more focused at watching the videos recorded by AIBUS and AIBUS provided the suspected nodules videos, it is plausible that HHUS might have missed some nodules when moving the probe.

The moderate agreement between HHUS and AIBUS diagnosed by senior radiologists was similar to other operator-independent automated breast scanners (11, 13, 14), indicating that AIBUS could provide clear images for breast cancer screening. In addition, AIBUS also had a shorter examination time than the traditional HHUS (in average 222.6 s for AIBUS and 236.1 s for HHUS) (9). For AIBUS, a nurse or health worker is required only to initiate the scan, and the machine then proceeds automatically with one click. This would reduce the costs for screening, address the shortage of radiologists, and increase the accessibility of breast cancer screening, especially for women in rural and remote areas.

To our knowledge, this is the first study comparing AIBUS and HHUS in their BI-RADS categorization. As the participated women were recruited in the breast cancer screening program in China with an age limit of 35–64, findings in this study cannot be generalized to elderly women. In addition, we did not detect malignant breast nodules in these women; therefore, further studies are still needed to compare their agreement in detecting malignant tumors. However, as AIBUS detected more breast nodules than HHUS, we speculate that AIBUS would probably detect more suspicious malignant lesions than HHUS, which might increase the sensitivity of breast cancer screening.

## Conclusion

In summary, we found that the results of AIBUS diagnosed by senior radiologists moderately agreed with that diagnosed by HHUS, and AIBUS detected more breast nodules. AIBUS could therefore be used in breast cancer screening in primary care setting with a shortage of local senior radiologists.

## Data availability statement

The raw data supporting the conclusions of this article will be made available by the authors, without undue reservation.

## Ethics statement

The studies involving human participants were reviewed and approved by the Ethics Committee in Fujian Maternity and Child Health Hospital. Written informed consent for participation was not required for this study in accordance with the national legislation and the institutional requirements.

## Author contributions

XH: conceptualization, funding acquisition, visualization, and writing—original draft. YQ and XH: data curation and validation. YQ: formal analysis. YQ, FB, JWa, and CL: investigation. YQ, XH, and YL: methodology. CL: project administration. YQ, YL, and JWu: resources. YQ and JWu: software. HY and XH: supervision. YQ, FB, JWa, CL, XH, HY, YL, and JWu: writing—review and editing. All authors contributed to the article and approved the submitted version.
